# Interspecies hybridization on DNA resequencing microarrays: efficiency of sequence recovery and accuracy of SNP detection in human, ape, and codfish mitochondrial DNA genomes sequenced on a human-specific MitoChip

**DOI:** 10.1186/1471-2164-8-339

**Published:** 2007-09-25

**Authors:** Sarah MC Flynn, Steven M Carr

**Affiliations:** 1 Genetics, Evolution, & Molecular Systematics Laboratory, Department of Biology, Memorial University of Newfoundland, St. John's, NL A1B3X9, Canada

## Abstract

**Background:**

Iterative DNA "resequencing" on oligonucleotide microarrays offers a high-throughput method to measure intraspecific biodiversity, one that is especially suited to SNP-dense gene regions such as vertebrate mitochondrial (mtDNA) genomes. However, costs of single-species design and microarray fabrication are prohibitive. A cost-effective, multi-species strategy is to hybridize experimental DNAs from diverse species to a common microarray that is tiled with oligonucleotide sets from multiple, homologous reference genomes. Such a strategy requires that cross-hybridization between the experimental DNAs and reference oligos from the different species not interfere with the accurate recovery of species-specific data. To determine the pattern and limits of such interspecific hybridization, we compared the efficiency of sequence recovery and accuracy of SNP identification by a 15,452-base human-specific microarray challenged with human, chimpanzee, gorilla, and codfish mtDNA genomes.

**Results:**

In the human genome, 99.67% of the sequence was recovered with 100.0% accuracy. Accuracy of SNP identification declines log-linearly with sequence divergence from the reference, from 0.067 to 0.247 errors per SNP in the chimpanzee and gorilla genomes, respectively. Efficiency of sequence recovery declines with the increase of the number of interspecific SNPs in the 25b interval tiled by the reference oligonucleotides. In the gorilla genome, which differs from the human reference by 10%, and in which 46% of these 25b regions contain 3 or more SNP differences from the reference, only 88% of the sequence is recoverable. In the codfish genome, which differs from the reference by > 30%, less than 4% of the sequence is recoverable, in short islands ≥ 12b that are conserved between primates and fish.

**Conclusion:**

Experimental DNAs bind inefficiently to homologous reference oligonucleotide sets on a re-sequencing microarray when their sequences differ by more than a few percent. The data suggest that interspecific cross-hybridization will not interfere with the accurate recovery of species-specific data from multispecies microarrays, provided that the species' DNA sequences differ by > 20% (mean of 5b differences per 25b oligo). Recovery of DNA sequence data from multiple, distantly-related species on a single multiplex gene chip should be a practical, highly-parallel method for investigating genomic biodiversity.

## Background

The development of DNA microarrays or "chips" has greatly increased the rate at which genomic data can be gathered. This highly-parallel technology has enabled thousands of genes or tens of thousands of single nucleotide polymorphisms (SNPs) to be recovered in a single experiment [1]. Most such arrays have been designed to assay variation within single species, such as *Drosophila melanogaster *[2] or humans, either among tissues (e.g., cancerous *versus *non-cancerous cell lines ([3,4], or among individuals that differ in some biomedically significant trait (e.g., obese *versus *non-obese patients [5]. But for evolutionary biologists, it is of greater interest to know how variation *among *species will be accommodated on species-specific microarrays. Consider two complementary questions. Can a microarray designed for the genome of one species recover accurate information on the genome of a closely-related species? Can a microarray that incorporates assays for homologous but distantly-related genes of different species successfully discriminate DNA from those species [6]?

The answers to these questions are of practical and theoretical interest. Evolutionary biologists are often interested in genetic relationships within and among more or less closely-related taxa of non-model organisms. Current costs of novel microarray design and execution of species-specific microarray experiments are prohibitive. Population and taxonomic studies would be more practical, if a common microarray design were useful over a broader range of taxa, say species within genera or closely-related families. Alternatively, if any given design is specific to a limited range of taxa, multiplex studies of more distantly-related taxa on the same microarray may be feasible. Here, we address the former question, and its implications for the latter, by measuring the ability of a species-specific DNA re-sequencing microarray to recover information from experimental DNAs over a wide range of sequence divergences.

DNA microarrays are commonly used to measure differential gene expression in cDNA libraries synthesized from mRNA transcriptomes, so as to determine which genes are active, where, and at what levels across experimental treatments (reviewed in [7]). Variant Detector Arrays (VDAs) measure not gene expression, but rather to variation in single-nucleotide polymorphism (SNP) among samples of interest [8]. VDAs rely on the ability of a ssDNA in the experimental sample to recognize and bind to its perfect oligonucleotide complement. A refinement of VDA microarrays is to evaluate, not just known SNPs, but all *potential *SNPs within a particular gene region. As developed by Affymetrix for their "GeneChip" protocols [9], a reference DNA sequence is represented on the microarray as a series of overlapping 25-base oligonucleotides ("oligos"), one for each position in the sequence. For each oligo, three additional variant oligos are included, each of which varies the central (13^th^) base. All possible SNP variants of a reference sequence of length *n *are thus represented on the microarray by a set of 4 × *n *oligonucleotides. An experimental sequence with any particular SNP variant in this quartet will hybridize with greatest fidelity to its exact complement, as indicated by the relative intensities of each of the probes bound at that position [10,11]. This procedure has been dubbed "resequencing," since it re-reads multiple homologous sequences in comparison with the reference sequence.

Over the past 25 years, studies of mtDNA have been extraordinarily successful in clarifying evolutionary relationships within and among species, due to a number of useful properties, including maternal inheritance, high rate of sequence evolution, and lack of recombination [11]. Gene order is broadly conserved across diverse vertebrate taxa. Recent comparative studies of multiple complete mtDNA genomes, both within [12,13] and among species [14,15], have demonstrated the power of genomics to investigate phenomena of intra- and interspecific population biology and evolution based on well-resolved, highly-corroborated gene trees [6]. The mtDNA genome has also been implicated in a number of human biomedical conditions [16,17].

One of the first applications of mtDNA to the study of evolution was an evaluation of the tempo and mode of molecular evolution of higher primates [18]. Studies of mtDNA and other genetic macromolecules have now established that the closest relatives of humans (*Homo sapiens*) are chimpanzees (*Pan *spp., including the Common Chimpanzee (*P. troglodytes*) and the Pygmy Chimp or Bonobo (*P. paniscus*)), with which we share a common ancestor ~5 MYBP [19]. The next closest relatives of chimps and humans are gorillas (*G. gorilla*), from which the chimp/human lineage diverged perhaps 7 MYBP [20]. Levels of mtDNA genome diversity vary among hominoid primate species, and are apparently lowest in *Homo *[21,12], due in part to our quite recent emergence "Out of Africa." Common Chimpanzees have a more polymorphic mitochondrial genome than humans, and variability within the Mountain Gorilla is as high as that between the two *Pan *species [22]. The greater diversity of apes in comparison with humans may be due to their historically more fragmented populations, differences in male and female migration, or directional selection [23]. There is now extensive interest in comparing the genetic material of humans and their closest relatives. The nuclear genome sequences of chimps and humans are more than 98% similar, and the focus of investigation is those differences that contribute to the uniqueness of the human species [24].

We investigated the efficiency and accuracy of microarray resequencing where experimental and microarray reference sequences are from different species, and the influence of the degree of sequence divergence on that performance. We use a human-specific mitochondrial DNA array [16] to resequence the homologous genomes of another human, as well as our two closest relatives, chimpanzee and gorilla, and a distant relative, Atlantic Cod (*Gadus morhua*). We compare these results to those obtained by conventional dideoxy sequencing. These experiments explore the limits of interspecies *in silico *hybridization, and in so doing contribute to the design and use of resequencing arrays for the study of intra- and interspecific population genomic evolution [6].

## Results

### Dideoxy reference sequences

**Figure 1:**
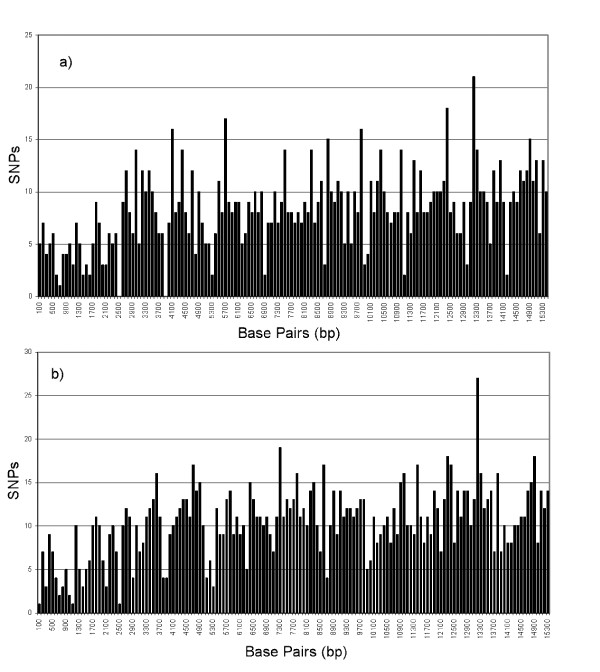
SNP density per 100 bps between the tiled human mtDNA sequence and the chimpanzee and gorilla mtDNA genomes, as identified by dideoxy DNA sequencing. SNP densities were calculated in a sliding window starting at Position 51 of the tiled sequence.

The reference microarray included 15452 bp of the revised Cambridge Reference Sequence (rCRS) [25], without the D-loop region. Dideoxy sequencing identified 1283 interspecific SNPs in the chimpanzee sequence as compared to this sequence. The sequence divergence is 8.21% between the two genomes. SNP density varied from 0 to 21 polymorphisms per 100 bases over the genome (Figure 1a). In the gorilla, there were 1600 single nucleotide polymorphisms, for an sequence divergence of 10.44% between the two genomes. As in the chimpanzee genome, SNP density was not uniform and varied from 1 to 22 per 100 bases (Figure 1b). SNP densities per 25 bp (the interval tiled by each oligo quartet) are given in Table 1. These range from 0 to 12, with a mode of 2 in both species.

**Table 1:** Percentage of 25-bp regions in the chimpanzee and gorilla mtDNA genomes that contain a given number of SNPs with respect to the tiled human mtDNA reference sequence on the MitoChip microarray

# SNPs SN	Chimpanzee	Gorilla
0	17.19	12.60
1	24.09	17.64
2	24.34	23.82
3	18.19	21.02
4	9.77	12.78
5	4.10	7.00
6	1.40	3.21
7	0.57	1.33
8	0.22	0.35
9	0.10	0.10
10	0.03	0.06
11	0.01	0.06
12	0.00	0.05

For the human comparison, we used an individual whose mtDNA genome sequence was known to differ from the tiled reference sequence by 32 SNPs (0.21% sequence divergence) in the region sequenced here [26].

### Comparison with microarray sequencing

Results of the chimpanzee and gorilla microarray sequencing experiments are presented as 2 × 2 tables in which calls of "SNP" or "non-SNP" by the microarray are classified as correct or incorrect in comparison with the known dideoxy reference sequence, which is taken as canonical. In the small number of cases where the dideoxy sequence traces might be regarded as ambiguous, the microarray confirmed the calls made *a priori*. We define *efficiency *as the overall proportion of bases called correctly by the microarray with respect to the *canonical *sequence, whether variable or not. We define *accuracy *as the proportion of known interspecific SNPs correctly identified with respect to the tiled microarray *reference *sequence Each of these four classes of calls may be further classified as of high or low confidence, for a total of eight classes (Table 2). Confidence in any call can be described as the ratio of the highest signal intensity to alternative signals, calculated as a differential signal-to-noise ratio (dS/N) as defined in Methods. We describe as "high-confidence" calls those made at dS/N greater than a designated cut-off. These may be either correct or incorrect ("errors"). For "low-confidence" calls, made with respect to a canonical sequence taken as a "null hypothesis", those that match that sequence are counted as correct, and those that do not are counted as "N". Considered without respect to such a sequence, all low-confidence calls are counted as "N".

**Table 2:** Efficiency, accuracy, and errors rates of microarray resequencing

	Correct	Incorrect
Microarray:	**High **+ *Low *confidence	Missed SNP +
SNP	at SNP site	*low-confidence N*
Microarray:	**High **+ *Low *confidence	Miscalled SNP +
no SNP	at non-SNP site	*low confidence N*

**Figure 2:**
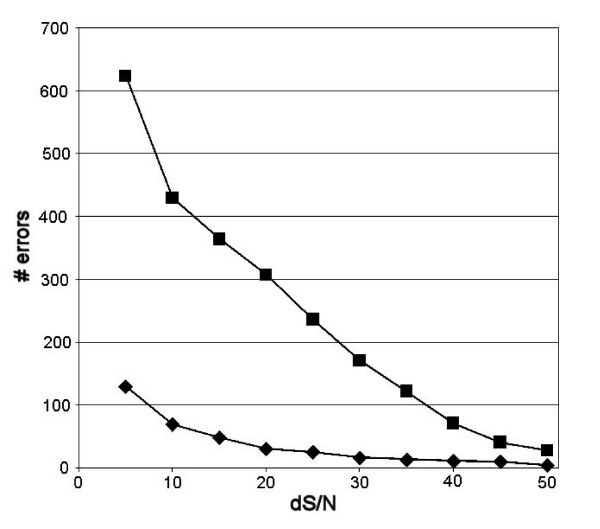
Number of errors at various dS/N cutoffs. The number of errors is the number of incorrect SNP identifications in chimpanzee (diamonds) and gorilla (squares).

*Efficiency *is the proportion of SNP and non-SNP sites identified correctly as compared with the canonical dideoxy sequence, either at high or low efficiency. *Accuracy *is the proportion of SNPs identified correctly. Correct and erroneous calls may be made at either **high **or *low *confidence, differentiated by dS/N= 0.20 (see Methods and Figure 2). The table is equivalent to a conventional 2 × 2 contingency table, with the categories on the second line transposed. This arrangement emphasizes the column totals of correct *versus *incorrect calls, and the assignment of low-confidence incorrect calls as *N*s, in Tables 3, 4, and 5.

The content and arrangement of the cells in these tables therefore differs from conventional 2 × 2 contingency tables, so as to emphasize the computation of correct, incorrect, and '*N*' calls. The inclusion of an 'N' category also makes a conventional ROC analysis problematic. It is important to appreciate that accurate identification of SNPs sites ('true positives') is a more important criterion of success than the total number of correct calls, including non-SNP sites ('true negatives'), because the latter do not contribute informative data to phylogenetic analysis. For example, given 1000 sites with 10 SNPs, the correct identification of all 10 SNPs along with 890 invariant sites and 100 'N's is a more desirable outcome than correct identification of 5 SNPs along with 5 SNP erros, 985 invariant sites, and 5 'N's, even though the conventional accuracy rates are 90% and 99%, respectively.

Among six human mtDNA genomes resequenced on a human-specific microarray, an empirical cut-off rule of dS/N = 0.13 allowed exclusion of all spurious SNPs, and correct identification of all known SNPs [26]. For the two primate resequencing experiments, we plotted the number of incorrect SNP calls made with various dS/N cut-offs between 0.05 and 0.50 (Figure 2). In *Pan*, error rates are < 1% at dS/N = 0.20 or greater, and are markedly greater at dS/N = 0.13 (0.61%) than at dS/N = 0.2 (0.31%). We therefore use dS/N = 0.20 as the cut-off for high- and low-confidence calls in this study.

### Efficiency and accuracy of microarray sequencing

With reference to the known dideoxy sequence, resequencing efficiency of the human mtDNA was 99.96%, including 98.44% and 1.52% of calls at high and low confidence, respectively (Table 3). All 32 known SNPs were identified, 24 at high confidence (100.00% accuracy).

**Table 3:** Efficiency, accuracy, and error of microarray resequencing of the human mtDNA genome (see table 2 for definitions)

Human	Correct	Incorrect
Microarray:	**24 **+ *8 *= 32	**0 **+ *0 *= 0
SNP	**0.16 **+ 0.04 = 0.20 %	*0 *%
Microarray:	**14705 **+ *714 *= 15419	**0 **+ *1*
no SNP	**95.17 **+ *4.62 *= 99.79	0 + *< 0.01 *= < 0.01 %
	**14729 **+ *722*	**0 **(0%)
	= 15451 (> 99.99 %)	+ 1 (< 0.01 %) *N*

With reference to their known sequences, resequencing efficiencies of the chimpanzee and gorilla mtDNAs were 85.07% and 88.45%, respectively (Tables 4 and 5). Of 1283 known chimpanzee SNPs, 72.49% were correctly identified (including 38.81% and 33.67% at high- and low-confidence, respectively), and 26.19% were errors at low-confidence that were counted as "N" s. There were 2.42% high-confidence errors. Of 1600 known gorilla SNPs, 67.19% were called correctly (46.50% and 20.69% at high-and low-confidence, respectively), and 19.13% were "N" s. There were 19.25% high-confidence errors.

**Table 4:** (a) Efficiency, accuracy, and error of microarray resequencing and (b) SNP density in the intervals ± 12 bp surrounding correct and incorrect calls of SNP and constant sites in chimpanzee mtDNA (see table 2 for definitions)

(a)	Correct	Incorrect
Microarray:	498 + *432 *= 930	**14 **+ *1940 *= 1954
SNP	**3.23 **+ *2.80 *= 6.03%	**0.09**+*12.56 *= 12.65%
Microarray:	**7290 **+ *4925 *= 12215	**17 **+ *336 *= 353
no SNP	**47.18 **+ *31.87 *=	**0.11 **+ *2.17 *= 2.28%
	**7788 **+ *5357*	**31 **(0.20%) errors
	= 13145 (85.07%)	+ 2276 (14.73%) *N*

(b)	Correct	Incorrect

Microarray:	**4.72, ***11.01*	**9.14, ***14.57*
SNP		
Microarray:	**3.93**, *10.96*	**6.44, ***15.48*
no SNP		

**Table 5:** (a) Efficiency, accuracy, and error of microarray resequencing and (b) SNP density in the intervals ± 12 bp surrounding correct and incorrect calls of SNP and constant sites in gorilla mtDNA (see table 2 for definitions)

(a)	Correct	Incorrect
Microarray:	**744 **+ 331 = 1075	**89 **+ 1170 = 1259
SNP	**4.81 **+ 2.14 = 6.96%	**0.58 **+ 7.57 = 8.15%
Microarray:	**9553 **+ 3040 = 12593	**219 **+ 30 *6 *= 525
no SNP	**61.82 **+ 19.67 =	**1.42 **+ *1.98 *= 3.40%
	**10297 **+ 3371	**308 **(1.99%) errors
	= 13668 (88.45%)	+ 1476 (9.55%) *N*

(b)	Correct	Incorrect

Microarray:	**8.64 + ***17.16 *=	**13.66 + ***17.63 *=
SNP	25.80%	31.29%
Microarray:	**7.73 **+ 13.47 =	**15.23 + ***17.11 *=
no SNP	21.20%	32.34%

Without reference to the known dideoxy sequences, as would be the case for *de novo *microarray sequencing of an unknown genome, low-confidence calls cannot be assigned *a priori *as either correct or incorrect. Then, in the chimpanzee, 50.40% of calls were correct at high-confidence, 49.40% were "N" s. There were 0.20% errors as before, and only 38.81% of SNPs were identified with high confidence. In the gorilla, 66.64% of calls were correct at high confidence, 30.37% were "N" s. There were 1.99% errors, and only 46.50% of SNPs were identified with high confidence.

The lower absolute efficiency and rate of SNP detection in the chimpanzee experiment as compared to that with the gorilla reflect lower probe intensities in the former. To compare interspecies efficiency and accuracy more directly, analysis of 6299 homologous positions called at high confidence in both species indicated a 0.41% error rate in the chimpanzee as compared with 2.11% in the gorilla. Of 379 chimpanzee SNP sites in this subset, 93.35% were correctly identified, as compared with 75.32% of 504 SNP sites in the gorilla genome Tables (6).

**Table 6:** Accuracy and error rate of microarray resequencing for 6299 bases called with high-confidence in both chimpanzee and gorilla

Chimpanzee	Correct	Incorrect
Microarray:	**365 **(5.79%)	**12 **(0.20%)
SNP		
Microarray:	**5908 **(93.80%)	**14 **(0.21%)
no SNP		
	**6272 **(99.59%) correct	**26 **(0.41%) errors

Gorilla	Correct	Incorrect

Microarray:	**406 **(6.45%)	**35 **(0.56%)
SNP		
Microarray:	**5760 **(91.44%)	**98 **(1.56%)
no SNP		
	**6166 **(97.89%) correct	**133 **(2.11%) errors

### Effect of SNP density on efficiency, accuracy, and probe intensity

In the chimpanzee experiment, correct high-confidence calls were made at a mean SNP density of 4.72% (i.e., 1.18 interspecific SNPs per 25 bps). Incorrect, high confidence calls occurred at SNP densities of 6.44 ~9.14 %. Correct, low-confidence calls occurred in regions with approximately 11% SNP density, above which, low confidence, incorrect calls occurred (Table 4). SNP densities in the gorilla followed the same general trends: correct high-confidence calls occurred at SNP densities < 8.64%. Incorrect, high-confidence calls occurred at SNP densities of 13.66 to 15.23%. Low confidence, correct calls occurred in regions with 13.47 to 17.16% SNP density, above which, low confidence, incorrect calls occurred (Table 5).

**Figure 3:**
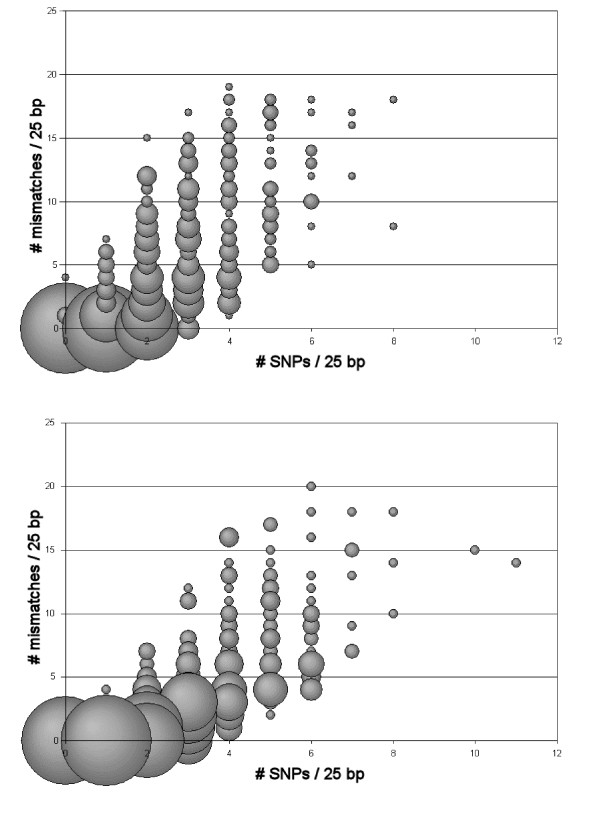
SNP density versus mismatch density per 25 bps in chimpanzee and gorilla mtDNA genomes. Bubbles are proportional to the number of events at each point.

Miscall rates ranged from 0 ~57 per 100 bps in the chimpanzee and from 0 ~44 per 100 bps in gorilla, the majority of which were found at low confidence in both experiments (Figure 3). The density of SNPs between the chimpanzee or gorilla and human genomes is positively correlated with the number of miscalls between these pairs. That is, as interspecific sequence divergence increases, so does the degree of miscalling and the errors observed in the microarray resequencing relative to the dideoxy sequencing.

### Resequencing of Atlantic Cod mtDNA

**Figure 4:**
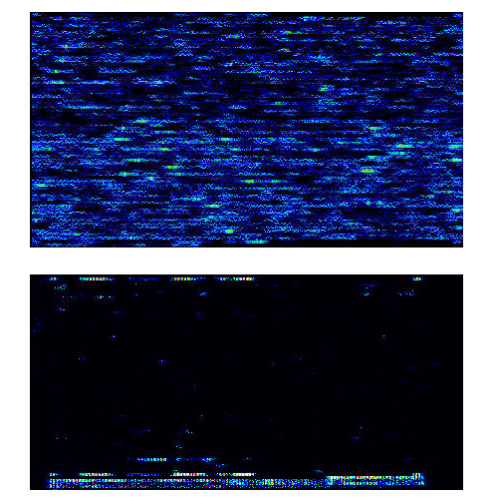
Experimental DNA binding of human and Atlantic Cod (*Gadus morhua*) mtDNA hybridized to a human-mtDNA-specific resequencing microarray.

Resequencing of mtDNA from Atlantic Cod (> 38% sequence divergence from human) on the human microarray generally produced extremely low probe intensities and dS/N values (Figure 4). The experiment identified 30 regions of > 12 contiguous bases called at high confidence, including one region of 120 bp that differed by only three mismatches between human and cod. The 592 bp correctly resequenced in these regions correspond to 3.83% of the cod genome.

## Discussion

### Efficiency and accuracy of interspecies resequencing

Microarray resequencing of human mtDNA sequences that differ by << 1% from the tiled human reference approach 100% efficiency and accuracy. Microarray resequencing of chimpanzee and gorilla DNA sequences, which differ by 8 and 10% from the tiled human sequence, recovers ~85% of those sequences, with < 2% error. Considered without respect to the known reference sequences, as would be the case if these were new individuals from the same species sequenced for the first time, efficiency of high-confidence sequence recovery falls to 67% in chimpanzee and to 50% in gorilla. Within this subset, overall error rates remain < 2%, however, accuracy of SNP identification falls from > 98% in chimpanzee to < 80% in gorilla.

**Figure 5:**
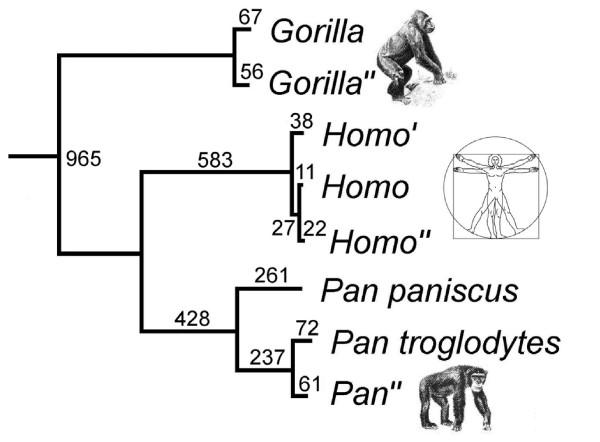
Phylogenetic relationships with and among *Gorilla*, *Pan*, and *Homo*, based on mitochondrial DNA genome sequences (without D-loops). The single minimum-length tree had a length of 2828. All nodes are supported in 100% of 10,000 bootstrap replications, Sequences marked (") are from the present paper. The unmarked sequences are from GenBank (*Gorilla *[NC_001645], *Pan troglodytes *[NC_001643], *P. paniscus *[NC_001644], and *Homo *[revised Cambridge Reference Sequence (rCRS): J01415.1]). The *Homo *sequence marked (') is from the individual (GenBank AF347008) identified in ref (12) as most divergent from the rCRS.

In resequencing closely-related humans whose pairwise sequence differences are << 1%, it is possible to adopt the null hypothesis that any site is invariant with respect to the tiled reference sequence, unless there is good evidence to the contrary. For example, among persons of European ancestry studied by Ingman et al. [12], the greatest pairwise difference in the 15452 bp region studied is 33 SNPs and is typically less than 20 (Figure 5). The dS/N can then be calculated as the difference between the strongest combined probe intensity and the reference base at any position [26]. Under these constraints, overall efficiency is extremely high, and high-confidence false-negative errors are rare to non-existent. Low-confidence false-positive errors are more common, the rate being determined by the dS/N criterion adopted. In contrast, there are 134 and 123 SNP differences, respectively, between the experimental chimpanzee and gorilla genomes sequenced here and their corresponding NCBI references [19], such that those references do not provide reliable null hypotheses. Then, one must rely on a confidence criterion to include or exclude putative SNPs, with consequent loss of information and/or accuracy

**Figure 6:**
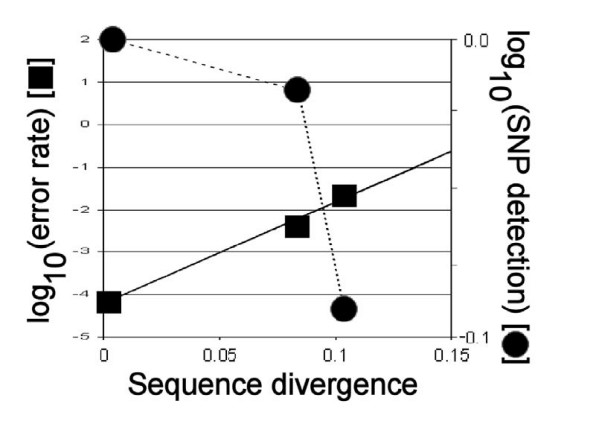
High-confidence error rate (E: squares) and SNP detection rate (circles) versus pairwise sequence divergence (D) for human, chimpanzee, and gorilla mtDNA genomes. The equation of the trend line is *log*(E) = (19.6)(D) – 3.9.

Decline in efficiency is log-linear with respect to sequence divergence (Figure 6). Extrapolation of the curve indicates that efficiency of sequence recovery would approach zero at ~20% observed sequence divergence. Such a divergence is typical of inter-ordinal or inter-class comparisons among vertebrate animals [27]. Some regions of the mtDNA are highly conserved evolutionarily, e.g., the 12S and 16S rDNA genes [28]. The cod resequencing experiment identified one contiguous tract of 120 bp in the 16S rDNA locus, within which there are only three nucleotide substitutions between primates and fish. These regions appear to be monomorphic within species or among closely related species [29].

Thus, microarray resequencing of experimental DNA genomes that diverge on average less than ~10% from the reference is able to recover a large part of the target sequence correctly. However, many of these calls are made at low confidence. The error rate is relatively low, but errors are more common at interspecies SNP sites than elsewhere, and the error rate increases sharply with the small added sequence divergence from chimpanzee to gorilla. Errors occur more or less uniformly over a wide range of probe intensities and confidence values. An increase in the stringency of the confidence criterion beyond a certain point does not increase accuracy, and only excludes more of the data (Figure 2).

### Influence of SNP density on efficiency and accuracy

In a microarray experiment, the presence of a SNP in an experimental sequence affects not only its binding to the oligo quartet tiling the corresponding position, but also to the 24 additional quartets in the 12 bp on either side of the SNP position. Among these 100 oligos, only one will match the target perfectly, 27 will mismatch at one position (three because of the chip design, 24 because of the SNP), and 72 will mismatch at two positions. Thus, reduced probe binding strength is expected on either side of a SNP, even at invariant sites. We typically observed Ns within a few bp of isolated SNPs. In human mtDNA, SNPs are typically spaced at 100s of bp with respect to the tiled reference, and are frequently associated with runs of lower-confidence Ns [26]. Regions in the ape genomes where SNPs are spaced > 25 bp apart are also associated with Ns, and are typically called correctly and at high confidence. Runs of Ns are associated with interspecific SNPs among higher primates resequenced on a human nuclear BRCA-specific microarray [30], where the SNP density is much lower than in mtDNA [31].

Where two SNPs occur within the 25 bp region covered by a particular SNP-specific oligo quartet, probe binding is affected at intermediate invariant positions. The pattern is specific and predictable. Consider two SNPs at an interval of 25 b, where one oligo quartet tiles one invariant position exactly 12 bp from either SNP. All four oligos in this set will have mismatches at their terminal (1^st ^and 25^th) ^positions, and three of four have an internal mismatch at the central (13^th) ^position. Binding and probe intensity will be severely reduced by these two or three mismatches, in comparison to the two adjacent positions, where only one or two mismatches occur. Where SNPs are spaced 13 <*n # *25b apart, the interference will extend to [(2)(25 - *n*) + 1] oligo quartets tiling the intermediate positions ("Flynn' s Rule"). In the ape data, we typically observe low probe intensity at all positions between two SNPs that occur within 25b. Precise patterns for any given oligo will be influenced by other factors, such as [G+C] content and distribution.

Multiple SNPs in the 25b region tiled by the oligo quartet further destabilize binding, and extensive tracts of Ns and miscalls are common. The data in Tables 4 &5 suggest some general guidelines, when SNP densities are expressed as expected numbers of SNP differences between experimental DNA and tiled oligo target, as in Table 1. High-confidence, accurate calls occur where the experimental sequence differs from the tiled array by 1 ~2 SNPs per 25b oligo, as is the case for about 67% and 54% of chimpanzee and gorilla genomes, respectively. At differences of 3 ~4 SNPs/oligo, high-confidence incorrect calls are common, which result in positive misidentification of SNPs; 28% and 34% of the chimpanzee and gorilla genomes fall in this category. At these densities, there is still sufficient sequence similarity for some probe-target hybridization to occur, although not always with accurate results. Where there are 5 SNPs/oligo or more (that is, when the experimental DNA differs from the reference oligos by an average of 20%), the decreased homology prevents binding with sufficient fidelity to discriminate accurately among SNP-specific oligos and generate high-confidence calls. This matches the prediction from Figure 6. The remaining 6% and 12% of the two ape genomes are at least as divergent as this from human. In cod, where an average of > 9 SNPs/oligo are expected, more than 95% of the cod mtDNA genome binds weakly if at all to the human-specific microarray.

## Conclusion

### Multi-species resequencing: implications for the "ArkChip"

For the primate genomicist, the optimum result of these experiments would have been efficient and accurate interspecific probe-target annealing with performance identical to that obtained within species. However, the goal of the present experiments was not to recover the chimpanzee, gorilla, or cod sequences, but rather to ascertain the limits of specificity of the human microarray. For the non-primate genomicist, the desirable result would be a complete failure of heterologous DNA to anneal to the human microarray. In the case of fish mtDNA, this is very nearly achieved (Figure 4). This "failure" indicates that it should be possible to tile both mammal and fish mtDNA genomes on the same microarray, apply a mixed pool of both species= DNAs to the chip, obtain species-specific annealing, and generate efficient and accurate sequences of both, simultaneously.

This is the essential idea behind the ArkChip. Using the new generation of microarrays that accommodates > 120 Kbp of reference sequence, we have designed a multispecies tiling that includes the complete forward and reverse sequences of the mtDNA genomes (including Control Regions) of three mammal species in different orders, three ray-finned fish species in different subclasses, and one bird species. Minimum interspecific divergence for these comparisons is > 23% ([6]. Experiments show that species genomes in two- and four-taxon combinations, from different orders and classes, are successfully and accurately sequenced (A. T. Duggan and S. M. Carr, work in progress).

Although the gorilla was only ~2% more divergent from human than the chimpanzee, the corresponding 3 ~4-fold increase in SNP identification errors indicates that this degree of divergence is at or beyond the limit the useful limits of interspecies microarray sequencing. The log-linear trend line suggests extinction of usable probe annealing at 15 ~20% divergence. It will be useful to define this empirically. For this purpose, our next closest primate relatives are orang-utans (*Pongo*: ~14% mtDNA sequence difference) and gibbons (*Hylobates*: ~17% difference) [19], followed by Old World Monkeys (Cercopithecidae, inc. *Papio*: ~25% difference) [32]. At the other end of the scale, mtDNA from our ancient cousins, such as *Homo neanderthalensis *[33]), might provide information as to how microarrays perform at less than 8% divergence. Alternatively, given the multispecies ArkChip, the three species of Atlantic wolffish (*Anarhichas*) [28], caribou and reindeer (New and Old World *Rangifer*, respectively) [34], and various cod species (*Gadus*) [15] all provide pairs that are only a few percent divergent.

## Methods

### Sources of DNA

Primate DNA was obtained from the roots of ten hairs plucked from a live chimpanzee (*Pan troglodytes*) at the Jardin Zoologique du Quebec, and from frozen heart tissue from a Western Lowland Gorilla (*Gorilla gorilla*) in the collection of the Royal Ontario Museum. DNA extractions were done with the QIAGEN QIAamp DNA Mini Kit Tissue Protocol. DNA from an Atlantic Cod (*Gadus morhua*) was purified by similar means.

### Dideoxy sequencing

The mitochondrial genomes were enzymatically amplified by a combination of standard and long-range, high-fidelity PCR methods, so as to isolate larger fragments [29], which minimizes subsequent variation in pooling amplicons for microarrays analysis. Primers designed for human mitochondrial DNA [12,26] that showed a high degree of homology with gorilla and chimpanzee reference sequences in GenBank were used for long-range amplification. Long-range amplification of the chimpanzee genome was done with three pairs of primers: h01F and h10R (for coding regions 12S to COX1), h09F and h13R (COX1 to COX3), and h14F and h17R (COX3 to ND4), for which the expected amplicon sizes were 6.7, 3.6, and 2.9 kb, respectively. Long-range amplification of the gorilla mtDNA genome was done with three pairs of primers: h01F and h06R (12S to ND2), h06F and h09R (ND1 to COX1), and h14 to h17 (COX3 to ND4), for which the expected amplicon sizes were 4.0, 2.9, and 2.9 kbp, respectively. Regions that remained outside the long-range amplicons were amplified by standard PCR methods. To generate reference sequences for the resequencing experiments, dideoxy sequencing was performed on an ABI 377 fluorescent sequenced as previously described [29], with the human- and/or gorilla-specific primers (Table 7). The chimpanzee and gorilla sequences were submitted to Genbank and assigned the accession numbers EU095335 and EU095336 respecively. The mtDNA sequence of an Atlantic Cod (*Gadus morhua*) was obtained by similar means [15,6].

**Table 7:** Sequence and positions of primers used to amplify and/or sequence the mtDNA genomes of chimpanzee and gorilla

**Primer**	**Sequence (5'-> 3')**	**PCR**	**5' Pos**
h01	F: CTCCTCAAAGCAATACACTG R: TGCTAAATCCACCTTCGACC	x	1 839
h02	F: CGATCAACCTCACCACCTCT R: TGGACAACCAGCTATCACCA		635 1 435
h03	F: GACTAACCCCTATACCTTCTGC R: GGCAGGTCAATTTCACTGGT		1 240 2 097
h04	F: AAATCTTACCCCGCCTGTTT R: AGGAATGCCATTGCGATTAG		1 889 2 773
h05	F: TACTTCACAAAGCGCCTTCC R: ATGAAGAATAGGGCGAGGG		2 558 3 388
h06	F: TGGCTCCTTTAACCTCTCCA R: AAGGATTATGGATGCGGTTG	x x	3 185 4 087
h07	F: ACTAATTAATCCCCTGGCCC R: CCTGGGGTGGGTTTTGTATG		3 874 4 851
h08	F: CTAACCGGCTTTTTGCCC R: ACCTAGAAGGTTGCCTGGCT		4 646 5 458
h09	F: GAGGCCTAACCCCTGTCTTT R: ATTCCGAAGCCTGGTAGGAT	x x	5 243 6 069
h10	F: CTCTTCGTCTGATCCGTCCT R: AGCGAAGGCTTCTCAAATCA	x x	5 858 6 742
h11	F: ACGCCAAAATCCATTTCACT R: CGGGAATTGCATCTGTTTTT		6 537 7 522
h12	F: ACGAGTACACCGACTACGGC R: TGGGTGGTTGGTGTAAATGA		7 316 8 224
h13	F: TTTCCCCCTCTATTGATCCC R: GTGGCCTTGGTATGTCCTTT	x x	8 010 8 824
h14	F: CCCACCAATCACATGCCTAT R: TGTAGCCGTTGAGTTGTGGT	x	8 619 9 557
h15	F: TCTCCATCTATTGATGAGGGTCT R: AATTAGGCTGTGGGTGGTTG		9 375 10 266
h16	F: GCCATACTAGTCTTTGCCGC R: TTGAGAATGAGTGTGAGGCG		11 061 10 919
h17	F: TCACTCTCACTGCCCAAGAA R: GGAGAATGGGGGATAGGTGT	x	10 703 11 503
h18	F: TATCACTCTCCTACTTACAG R: AGAAGGTTATAATTCCTACG	x	11 337 12 201
h19	F: AAACAACCCAGCTCTCCCTAA R: TCGATGATGTGGTCTTTGGA	x x	11 959 12 934
h20	F: ACATCTGTACCCACGCCTTC R: AAGGGGTCAGGGTTCATTC	x	12 727 13 694
h21	F: GCATAATTAAACTTTACTTC R: AGAATATTGAGGCGCCATTG		13 489 14 425
h22	F: TGAAACTTCGGCTCACTCCT R: AGCTTTGGGTGCTAATGGTG	x	14 245 15 405
h23	F: TCATTGGACAAGTAGCATCC R: GAGTGGTTAATAGGGTGATAG	x x	15 200 16 009
Gg11	F: CCCACACAGTTTATGTAGCTTACCTC	x	6612
Gg12	R: GAATATTAGCTTTGGGTGCTGATGGTGG	x	8093
Gg18	F: CTATCCCTCAACCCCGATATTACT R: CTTAACCAACTACAACCCCAGACTC	x x	11 520 12 109
Gg20	F: CCTTACTTCAACCTCCCTAGCCATTG R: CGTTAACTACTCCTTCCGCCAACTCC		12 859 14 353

### Microarray resequencing

We used a commercial MitoChip microarray (Affymetrix) to resequence a 15452 bp of the coding portion of the human mitochondrial DNA (mtDNA) genome, excluding the CR control region and including two rDNA, 22 tDNA, and 13 protein-coding genes [16]. These features are tiled both as the heavy and light strands (designated "sense" and "antisense") strands, such that every base is assayed twice. To fill up the balance of the available 30 Kb feature array, the MitoChip includes duplicate tiling of this portion of the mtDNA genome, without the 12S and 16S rDNA genes. For these 12805 positions, there are thus a total of four replicates.

Preparation for microarray resequencing includes pooling of amplicons at equimolar concentration, nuclease fragmentation, labeling, and fluorescent staining, according to the Affymetrix GeneChip CustomSeq resequencing Array protocol, v. 2 (2003). PCR amplicons were first pooled, such that each nucleotide was present in equimolar quantities. Calculations were based on known amplicon size and concentration as determined with an Eppendorf BioPhotometer. Appropriate volumes of each amplicon were added to a single pool (Table 8), which was then brought to dryness in a vacuum centrifuge and reconstituted in buffer. Pooled DNA was fragmented, to produce DNA fragments of uniform small size (ca. 20 ~200 bp). Fragmented DNA was labeled with a poly-A tail via an rTdT transferase reaction. The labeled fragments were applied to the GeneChip microarray and hybridization proceeded for 20 hrs. Each array was stained with a fluorescent SAPE stain in a GeneChip Fluidics Station according to protocol. The arrays were scanned with the Affymetrix GeneChip Scanner 3000 and analyzed with the GeneChip DNA Analysis Software.

**Table 8:** Properties of PCR amplicons used for microarray resequencing of chimpanzee and gorilla: size, required mass and observed concentration, and volume added to pool

Chimpanzee PCR Amplicons	Size (bp)	Mass (ng)	Conc. (ng/mL)	Volume (uL)
h01F, h10R	6736	244.5	19.8	12.35
h09F, h13R	3582	130.0	11.1	11.71
h14F, h17R	2885	104.7	5	20.95
h18F, h18R	866	31.4	9.4	3.34
h19F, h19R	976	35.4	6.9	5.13
h20F, h22R	2679	97.2	12.5	7.78
h23F, h23R	807	29.3	11.1	2.64

Gorilla PCR Amplicons	Size (bp)	Mass (ng)	Conc. (ng/mL)	Volume (uL)

h01F, h06R	4082	148.2	19.4	7.64
h06F, h09R	2888	104.8	15.1	6.94
h10F, h10R	886	32.2	14.1	2.28
Gg11F, Gg12R	1420	51.5	14.7	3.51
h13F, h13R	815	29.6	20.6	1.44
h14F, h17R	2885	104.7	22.8	4.59
Gg18F, Gg18R	590	21.4	25.4	0.84

### Numerical Analysis

Experimental results from chimpanzee, gorilla, and cod were assembled with those from a human [26]. Output from each array experiment consisted of eight sets of probe intensity values, corresponding to the A, C, G, and T oligonucleotide variants of the sense and antisense strands at each of 15,452 tiled positions. Elaborate scoring algorithms based on likelihood methods have been developed [35]. We applied a simpler arithmetic algorithm as follows. Sense and antisense probe intensities were summed to give four base-specific intensity scores for each position, and the highest and second- highest scores for each position were identified, along with the sum of intensity scores across all four bases. The difference between the two highest intensities was divided by the sum, which yielded a value defined as the differential signal-to-noise ratio (dS/N). This value expresses the confidence placed on each call. The approach is similar to those used previously [30], except that it includes standardization for total probe intensity.

Comparison of the probe intensity values and dS/N scores for the partially duplicated region of the genome on the microarray shows them in all cases to be virtually identical to the main series [results not shown].

Distribution of SNP density between the ape dideoxy sequences and the tiled human sequence was calculated as a sliding window of 25 bp, starting at Position 13 of the tiled sequence. The numbers of interspecific SNPs versus intraspecific miscalls (positions where the microarray call differed from the dideoxy sequence) within each of the two primate sequences were compared in a sliding window of 25 bp extending 12b on either side of each position, starting at Position 13 of the tiled reference. The SNP versus mismatch densities were averaged over all calls in each of the eight classes of Table 2.

### Phylogenetic Analysis

To compare intra- and interspecific differentiation among the mtDNA genomes, we performed a phylogenetic analysis with the program PAUP (36). We aligned the 15,452b of the three primate dideoxy sequences in this paper, together with the homologous portions of five additional sequences from GenBank listed in Figure 5. We performed a branch-and-bound search, with all positions weighted equally. The tree was rooted with *Gorilla *as the outgroup to *Pan *and *Homo*.

## Competing interests

The author(s) declares that there are no competing interests.

## Authors' contributions

The experiments described were part of the BSc (hons) thesis research of SMCF, including dideoxy sequencing and preparation and execution of the microarray experiments. SMC supervised the research in his lab, developed the algorithms for the numerical analysis, and drafted the manuscript. Both authors read and approved the final version of the manuscript.
